# Improving awareness of preconception health among adolescents: experience of a school-based intervention in Lebanon

**DOI:** 10.1186/1471-2458-14-774

**Published:** 2014-07-31

**Authors:** Lama Charafeddine, Rym El Rafei, Sophie Azizi, Durriyah Sinno, Kawthar Alamiddine, Christopher P Howson, Salimah R Walani, Walid Ammar, Anwar Nassar, Khalid Yunis

**Affiliations:** the National Collaborative Perinatal Neonatal Network, American University of Beirut Medical Center, Beirut, Lebanon; American University of Beirut Medical Center, Beirut, Lebanon; March of Dimes Foundation, White Plains, New York U.S; Director General of the Ministry of Public Health of Lebanon, Beirut, Lebanon; Department of Pediatrics and Adolescent Medicine, American University of Beirut Medical Center, Beirut, Lebanon

**Keywords:** Preconception, Adolescents, Awareness campaign, Health education program, Health information

## Abstract

**Background:**

Maternal behavior before and after conception affects maternal and child health. Limited awareness of adolescents in preconception health may be addressed through school education. The aim of this intervention is to assess preconception health awareness among adolescents in Lebanese high schools and to test the effectiveness of a one-time educational session in improving preconception knowledge.

**Methods:**

The intervention consisted of a 30-minute educational session about good practices in preconception health, developed by the National Collaborative Perinatal Neonatal Network’s (NCPNN) research team. A convenience sample of high school Lebanese students in grades 10 to 12, aged 14 to 26 years old, from 70 private and public schools in all six Lebanese provinces, participated in the intervention in 2011 and 2012. A multiple-choice questionnaire administered prior to and 2 months after the session was used to assess knowledge improvement among the students.

**Results:**

A total of 7,290 students were enrolled. After the session, mean scores of correct answers increased from 4.36 to 6.42 out of 10, representing a 47.2% improvement (*p* < 0.001). The percent of correct answers increased for all the questions regarding health practices (*p* < 0.001). The greatest improvement was observed for questions about Trisomy 21, folic acid intake and toxoplasmosis with percentages improvement of 96%, 172% and 83% respectively. Being female or in private school was a significant predictor of higher scores in both pre-test and post-test (*p* < 0.001).

**Conclusions:**

Awareness campaigns in schools increased the preconception health knowledge among high school students. We recommend expanding the scope of this intervention into universities in Lebanon.

**Electronic supplementary material:**

The online version of this article (doi:10.1186/1471-2458-14-774) contains supplementary material, which is available to authorized users.

## Background

Current scientific evidence indicates that, improving a woman’s health before pregnancy (preconception health and health care) maximizes the potential for a healthy pregnancy outcome for both mother and infant [1]. Many women enter pregnancy in poor health with untreated preexisting conditions or without awareness of healthy behaviors, such as taking a daily folic acid supplement, avoiding exposure to tobacco and other teratogenic agents or updating their immunizations; all of which put them at risk for maternal and neonatal adverse outcomes [2–4]. In addition, millions of women remain at risk for unintended pregnancy and lack the knowledge or motivation for access to reproductive health services [5].

Preconception health care involves the provision of health care to women and men during their reproductive years [6] and offering evidence-based interventions to reduce adverse outcomes in future pregnancies [7, 8]. Adolescence is a particularly important point in the reproductive, maternal, newborn and child health (RMNCH) continuum to promote preconception health [9]. Educating young women about healthy lifestyles not only empowers them as individuals in their own right, but also it can result in healthier maternal and newborn outcomes should they become pregnant [10–12].

Evidence from Lebanon highlights a need for promoting preconception health awareness. In a national survey of married Lebanese women aged 18 to 45 years, Nasr et al. found that 40% of the participants had not heard about pre-pregnancy folic acid and its prevention of neural tube defects (NTD) [13]. Tamim et al. showed a low prevalence (14%) of preconception folic acid intake among 5,280 Lebanese pregnant women [14].

National data from the recently established birth defects surveillance system showed that among the mothers of 170 newborns identified with NTD in one year, only 6.7% reported having taken folic acid prior to pregnancy (unpublished data).

In addition, the 2011 “Global Youth Tobacco Survey in Lebanon” reported that among 2,339 male and female youths surveyed, 59.2% had never smoked cigarettes or water-pipe (shisha) and 11.3% currently smoke cigarettes; of those surveyed, 70% were between 13 and 15 years of age [15]. Other growing problems during pregnancy are obesity and diabetes which are both associated with adverse maternal and neonatal outcomes [16, 17]. In the Lebanese adolescent population, the prevalence of obesity was reported as 28% and 31.5% in 1997 and 2009 respectively [16]. Furthermore, reproductive outcomes are also linked to the father’s health and lifestyle; [18] whereas fathers’ smoking leads to fetal secondhand smoke exposure, hence increased risk of low birthweight and later, sudden infant death syndrome [19, 20]. Thus, it is essential to target young men’s awareness about preconception health and behaviors and encourage them to play a more supportive role prior to and during their partner’s pregnancy [21].

Improving health knowledge, attitudes and behaviors of both young women and men is an essential step in promoting preconception health [6]. The school system represents a natural venue to reach adolescents. Indeed, college health education programs have been shown to improve preconception awareness among youth [21, 22].

The purpose of this paper is to present the experience of a preconception health education intervention conducted in high schools in Lebanon, and to demonstrate the impact of such a program in improving knowledge of benefits of preconception health.

## Methods

### Study, design and setting

This intervention was carried out in 70 public and private high schools in Lebanon over a 2-year period (2011 to 2012) by the National Collaborative Perinatal Neonatal Network (NCPNN) team in collaboration with the Lebanese Ministries of Education and Health. The NCPNN is a hospital-based network of health professionals across Lebanon whose goal is to improve maternal and neonatal health [23].

The intervention was implemented in all six Lebanese provinces: the Capital city, the central, southern, northern, eastern and east-southern provinces. It consisted of a 20-minute lecture session for students in grades 10 through 12. The session material, developed by experts from the NCPNN team, included 28 slides addressing conditions affecting reproductive outcomes. These conditions were: obesity and underweight; infectious diseases, in particular toxoplasmosis; chronic conditions such as diabetes and epilepsy, smoking, alcohol and recreational drug usage as well as certain medications. At each session, the importance of a healthy lifestyle, childhood immunizations and pre-pregnancy folic acid intake were stressed. Birth defects, preterm births and their increased risks associated with poor preconception health status were among the topics presented.

### Process and intervention

Exemption approval was obtained from the Institutional Review Board of the American University of Beirut to conduct the intervention. Consent by the students was not needed as the knowledge questionnaires didn’t include any identifiers and it was specified verbally to them that their participation to fill in the questionnaires was voluntary. Written approval from the Lebanese Ministry of Education was however obtained. A list of public schools was provided by the Lebanese Ministry of Education accordingly. An enforcement letter for participation addressed to the schools’ administrators and teachers was sent by the Lebanese Ministry of Education to each public school. Following permission from the schools’ administrators of both public and private schools, the implementation visits were scheduled. Verbal informed consent also was taken from the students upon distribution of the anonymous pre-test questionnaire. Refusal to fill in the pre-test questionnaire did not hamper the students from attending the lecture.

The intervention was completed in 41 public schools chosen randomly from the list provided from the Lebanese Ministry of Education to cover all provinces. Following the convenience sampling, 49 private schools from all provinces were approached; 29 agreed to participate.

Before initiating the intervention, the NCPNN lecturers (pediatricians, obstetricians, nurse leaders) discussed the presentation’s content and reached an agreement regarding the uniformity of leading each session. Prior to each session, the project leader reviewed again the presentation with the lecturer for that session. On average, two schools were visited per day over a 3-month period. Students from grades 10 to 12 were selected to attend the lecture.

To assess students’ baseline knowledge, a self-administrated questionnaire was developed in English and Arabic and was given to each participant before the session (Additional file 1). The questionnaire, which took about 10 minutes to complete, had two sections. The first section asked about socio-demographic characteristics; the second section contained ten multiple choice questions about the preconception health topics of discussion. Students were informed of their right to decline to answer individual questions; they were reassured that the questionnaire would be anonymous and that their performance would neither affect their grades nor cause any penalty for wrong answers.

After administering the questionnaire, the NCPNN lecturer gave the presentation in Arabic or English depending on the students’ preference. The session continued with an informal open discussion in which students were given the opportunity to ask further questions. Two months after the intervention, the same questionnaire was mailed to each school requesting to have it completed by the same students who participated in the educational sessions and returned by prepaid mail.

### Statistical analysis

Data were analyzed using Statistical Program for Social Science version-20 (SPSS). A calculated variable “total score of correct answers” was created to measure each student’s knowledge level on a scale from 1 to 10, based on the responses to the 10 knowledge questions. Correct answers were scored as “1” and incorrect or “I do not know” answers were scored as “0”. Means and standard deviations were calculated for continuous variables while the categorical variables were analyzed in frequencies and percentages. Chi-square test was used to compare categorical variables and independent sample t-test to compare total scores. Multiple linear regression was used to assess the independent relationship of the intervention and all the socio-demographic characteristics on all scores (pre and post). Statistical significance was set at an alpha of 0.05.

## Results

### Study population

The health education intervention was conducted in 70 schools (41 public and 29 private) reaching a total of 7,290 students. Overall, 79.4% of those who took the pre-test returned the post-intervention questionnaires 2 months after their session.

Of the total participants at baseline, 63.6% were from public schools, 57.1% in grade 11, 75.0% were aged between 14 and 18 years, 18.9% were boys, and 26.3% were engaged to be married in the year following the intervention. Information about the students’ parental profession showed that the majority of mothers were housewives, and only 4.1% of the fathers were working in the medical or paramedical field (Table 1).

**Table 1:** Socio-demographic characteristics of the students based on pre-test analysis*

General characteristics of the students	N = 7,290 (%)
**Gender**	
Male	1,346 (18.9%)
Female	5,791 (81.1%)
**Age**	
<18 years	5,198 (75.0%)
≥18 years	1,734 (25.0%)
Mean ± SD	16.94 ± 0.99
**Provinces**	
Capital city (Beirut)	1,323 (18.1%)
Central Province (Mount Lebanon)	1,600 (21.9%)
Southern Province (South Lebanon)	1,916 (26.3%)
Northern Province (North Lebanon)	1,189 (16.3%)
Eastern Province (Bekaa valley)	528 (7.2%)
East southern province (Nabatieh)	734 (10.1%)
**School type**	
Public	4,639 (63.6%)
Private	2,651 (36.4%)
**Study class**	
Grade 10	397 (5.7%)
Grade 11	3,996 (57.1%)
Grade 12	2,561 (36.6%)
Technical baccalaureate 3 (BT3)	41 (0.6%)
**Parents with chronic condition**	
No	5,091(75.1%)
Yes	1,470 (21.7%)
Don’t know	218 (3.2%)
**Father’s profession**	
Not working	158 (2.6%)
Technical & other	2,382 (39.2%)
Non-medical	3,288 (54.1%)
Medical & paramedical	246 (4.1%)
**Mother’s profession**	
Housewife	4,673 (78.1%)
Technical & other	235 (3.9%)
Non-medical	924(15.4%)
Medical & paramedical	149 (2.5%)
**The following characteristics were from the 2** ^**nd**^ **year of the intervention only**	**N = 3697, (%)**
**Marital status**	
Single	2,576 (73.7%)
Engaged	920 (26.3%)
**Father’s education**	
Primary or less	456 (13.2%)
Middle school	964 (27.9%)
Secondary/high school	966 (28.0%)
University	1,070 (31.0%)
**Mother’s education**	
Primary or less	341 (9.8%)
Middle school	965 (27.7%)
Secondary/high school	1,203 (34.5%)
University	980 (28.1%)

*Missing not shown.

### Knowledge assessment

The participants’ mean knowledge score at baseline was 4.36 out of 10. Two months after the session, mean knowledge scores in the post-test increased to 6.42 (*p* < 0.001), representing 47.2% improvement. The percentage of students who answered correctly improved significantly for all the questions (*p* < 0.001). Answers to questions regarding risk of Trisomy 21, folic acid intake and toxoplasmosis showed the highest improvement percentages of 96%, 172% and 83% respectively (Table 2).

**Table 2:** Knowledge assessment score before and after the intervention*

Preconception health components	Pre-test N, (%) N = 7,290	Post-test N, (%) N = 5,786	***p*** -value
**Seeking medical advice**			
True	4,646 (65.2%)	4,243 (75.3%)	< 0.001
False	2,119 (29.7%)	1,262 (22.4%)	
Don’t know	361 (5.1%)	132 (2.3%)	
**Trisomy 21**			
True	1,785 (24.8%)	2,777 (48.8%)	< 0.001
False	4,058 (56.3%)	2,416 (42.4%)	
Don’t know	1,364 (18.9%)	502 (8.8%)	
**Pre-pregnancy folic acid**			
True	1,529 (21.4%)	3,240 (58.3%)	< 0.001
False	2,751 (38.6%)	1,406 (25.3%)	
Don’t know	2,853 (40.0%)	908 (16.3%)	
**Diabetes**			
True	3,836 (53.5%)	3,963 (69.9%)	< 0.001
False	353 (4.9%)	736 (13.0%)	
Don’t know	2,977 (41.5%)	968 (17.1%)	
**Epilepsy**			
True	2,851 (40.3%)	3,265 (58.3%)	< 0.001
False	1,040 (14.7%)	1,103 (19.7%)	
Don’t know	3,192 (45.1%)	1,235 (22.0%)	
**Toxoplasmosis**			
True	3,130 (43.4%)	4,484 (79.4%)	< 0.001
False	2,000 (27.8%)	667 (11.8%)	
Don’t know	2,074 (28.8%)	497 (8.8%)	
**Medication & pregnancy**			
True	3,864 (54.5%)	4,420 (79.0%)	< 0.001
False	906 (12.8%)	491 (8.8%)	
Don’t know	2,319 (32.7%)	685 (12.2%)	
**Obesity**			
True	2,519 (35.1%)	3,388 (59.6%)	< 0.001
False	2,693 (37.6%)	1,728 (30.4%)	
Don’t know	1,956 (27.3%)	572 (10.1%)	
**Alcohol, smoking and illicit drugs**			
True	5,223 (72.3%)	4,804 (84.0%)	< 0.001
False	1,385 (19.2%)	652 (11.4%)	
Don’t know	616 (8.5%)	261 (4.6%)	
**Vaccination**			
True	2,399 (66.6%)	2,410 (86.1%)	< 0.001
False	329 (9.1%)	128 (4.6%)	
Don’t know	873 (24.2%)	262 (9.4%)	
**Total score (mean ± SD)**	**4.36 ± 1.8**	**6.42 ± 2.1**	**< 0.001**

*Missing not shown.

The number of correct answers was compared among male and female students in mixed classes only. The number of female students who answered correctly in the post-test was significantly higher than the males in all the topics (*p* < 0.012) except for “folic acid intake before pregnancy” (*p* = 0.10) and “alcohol, smoking and illicit drugs” (*p* = 0.45).

When answers were analyzed by grade level, scores at baseline were slightly different among grades 10, 11 and 12 with the highest score reached among grade 12 students (4.83 over 10). However, following the educational session, grade 10 students showed the highest knowledge improvement rate of 64.8% as compared to the other grades: 45.8% for grade 11 and 39.9% for grade 12. The number of correct responses in the post-test was significantly different among all three grades and for all questions (*p* < 0.02) except for the ones on toxoplasmosis (*p* = 0.44), medications & pregnancy (*p* = 0.23) and alcohol, smoking and illicit drugs (*p* = 0.13).

### Factors associated with higher test scores

Table 3 shows the relationship between the scores before and after the intervention and the socio-demographic characteristics at the bivariate level. The mean scores in both pre- and post-tests were statistically related to gender, age group, provinces, school type and grade level (*p* < 0.05).

**Table 3:** **Factors associated with knowledge scores for “Pre- and post-test” questionnaires**

	Pre-test		Post-test	
Socio-demographic characteristics	Mean ± SD	***p*** -value	Mean ± SD	***p*** -value
**Gender**				
Male	4.21 ± 1.92	< 0.001	5.91 ± 2.37	< 0.001
Female	4.41 ± 1.79		6.54 ± 2.06	
**Age**				
<18 years	4.35 ± 1.82	0.019	6.91 ± 2.14	< 0.001
≥18 years	4.47 ± 1.77		6.61 ± 2.12	
**Provinces**				
Capital city (Beirut)	4.30 ± 1.77	< 0.001	5.87 ± 2.12	< 0.001
Central Province (Mount Lebanon)	4.37 ± 1.79		6.37 ± 1.96	
Southern Province (South Lebanon)	4.57 ± 1.79		6.71 ± 1.98	
Northern Province (North Lebanon)	4.15 ± 1.83		5.69 ± 2.24	
Eastern Province (Bekaa valley)	4.31 ± 1.75		7.05 ± 2.12	
East southern province (Nabatieh)	4.33 ± 1.97		7.44 ± 2.17	
**School type**				
Public	4.33 ± 1.80	0.017	6.32 ± 2.11	< 0.001
Private	4.43 ± 1.84		6.56 ± 2.19	
**Grade level**				
Grade 10	4.16 ± 1.95	< 0.001	7.15 ± 2.14	< 0.001
Grade 11	4.33 ± 1.82		6.44 ± 2.17	
Grade 12	4.54 ± 1.75		6.41 ± 2.02	
Technical baccalaureate 3 (BT3)	4.34 ± 2.11		6.66 ± 2.19	
**Parents with chronic condition**				< 0.001
No	4.36 ± 1.79	< 0.001	6.43 ± 2.14	
Yes	4.52 ± 1.83		6.56 ± 2.06	
Don’t know	3.79 ± 1.94		5.16 ± 2.42	
**Father’s profession**				
Not working	4.29 ± 1.91	0.404	6.51 ± 2.30	< 0.001
Technical/other	4.33 ± 1.80		6.14 ± 2.10	
Non-medical	4.41 ± 1.81		6.57 ± 2.13	
Medical & paramedical	4.40 ± 1.69		6.34 ± 2.16	
**Mother’s profession**				
Not working	4.37 ± 1.81	0.139	6.55 ± 2.10	0.222
Technical/other	4.40 ± 1.73		6.30 ± 1.92	
Non-medical	4.47 ± 1.78		6.43 ± 2.22	
Medical & paramedical	4.64 ± 1.75		6.64 ± 1.97	

The independent factors predicting higher knowledge scores were: the intervention itself (β = 1.944, 95% CI 1.834 - 2.054); female gender (β = 0.421, 95% CI 0.309 - 0.532); the location by province (central province: β = 0.356, 95% CI 0.182 - 0.530, southern province: β = 1.009, 95% CI 0.844 - 1.173, northern province: β = 0.566, 95% CI 0.362 - 0.769, eastern province: β = 0.783, 95% CI 0.475 - 1.090, east-south province: β = 1.128, 95% CI 0.918 - 1.337); school type (β = 0.286, 95% CI 0.147 - 0.425); and grade level (Grade11: β = 0.221, 95% CI −0.017 - 0.458, Grade 12: β = 0.506, 95% CI 0.261 - 0.751, technical baccalaureate 3 (BT3): β = 0.931, 95% CI 0.406 - 1.455). Having participated in the intervention, being a female, going to a private school or living in the southern provinces were independently associated with a higher score of correct answers (p < 0.001) (Table 4).

**Table 4:** **Independent predictors of knowledge scores (N=5817)**

	B*	95.0% CI*	***p*** -value
**Intervention**			
Pre	1.0	Reference	
Post	1.944	1.834, 2.054	< 0.001
**Gender**			
Male	1.0	Reference	
Female	0.421	0.309, 0.532	< 0.001
**Provinces**			
Capital city (Beirut)	1.0	Reference	
Central Province (Mount Lebanon)	0.356	0.182, 0.530	<0.001
Southern Province (South Lebanon)	1.009	0.844, 1.173	< 0.001
Northern Province (North Lebanon)	0.566	0.362, 0.769	< 0.001
Eastern Province (Bekaa valley)	0.783	0.475, 1.090	0.001
East southern province (Nabatieh)	1.128	0.918, 1.337	< 0.001
**School type**			
Public	1.0	Reference	
Private	0.286	0.147, 0.425	< 0.001
**Grade Level**			
Grade 10	1.0	Reference	
Grade 11	0.221	−0.017, 0.458	0.069
Grade 12	0.506	0.261, 0.751	< 0.001
Technical baccalaureate 3 (BT3)	0.931	0.406, 1.455	0.001

In the following table, only mixed classes is included in the analysis.

*B: Regression coefficient, CI: confidence interval.

## Discussion

This study describes a successful implementation of a school-based intervention to increase Lebanese adolescents’ knowledge and importance about preconception health practices. Students in the higher grade levels had higher baseline knowledge compared to those in lower grades who demonstrated greater improvement from baseline. Being female or enrolled in a private school was a significant predictor of higher scores at baseline as well as after the intervention.

Intervention to educate adolescents about preconception health is novel in our region because of the sensitivity of the topic. Preconception health education is not part of the official school curriculum, and preconception issues are not discussed between parents and adolescents [24]. Pediatricians in Lebanon are less likely to address prevention and health education routinely in their practice (personal observation).

A similar intervention in rural India demonstrated an increase in adolescents’ knowledge after giving lectures on reproductive health; [25] Delgado showed moderate improvement in knowledge after preconception health courses at the University of Miami [21, 22].

In the present study, adolescents’ knowledge about the benefits of preconception health and in particular to the importance of folic acid intake prior to conception was particularly poor, and this was true for all students regardless of their age, gender or grade level. These results are in accordance with Delgado who found that only 32% of college students were aware of the importance of folic acid intake in the early fetal development [21]. Similarly, 36% of women in the undergraduate psychology program in Virginia Commonwealth University had not heard about folic acid [26].

Among the high school students who participated in our intervention, 26% were engaged to be married and were unlikely to be taking folic acid since there was a large gap in knowledge regarding the importance of folic acid prior to pregnancy. Preconception folic acid intake represents a major public health challenge since universal fortification in Lebanon has not been implemented, yet the rate of neural tube defects (NTD) in Lebanon is estimated at 13.2 per 10,000 (unpublished data from the national birth defects registry/NCPNN/ May 1^st^ 2012 – December 31^st^ 2013) compared to 4.6 per 10,000 reported in the United States [27].

Before attending the educational session, a modest percent of students were knowledgeable about the fetal-related risks of obesity (35.1%) and diabetes (53.5%). This finding is in agreement with another study where 65% of diabetic adolescents were unaware of preconception health and 34% knew nothing about diabetes and pregnancy [28]. Knowing that the prevalence of obesity among children in Lebanon has increased significantly over the past decade [16], it is important to increase adolescents’ awareness about the implications of obesity on the health of mother and fetus during pregnancy.

After participating in this intervention, approximately 80% of the high school students showed improvement in their knowledge scores in contrast to 100% among college students receiving a semester-long class intervention [22]. This finding could be related either to the younger age of the high school students or to factors related to the mode of delivery of such interventions.

It is interesting to note that after the intervention, students in lower grades had better scores than those in higher grades. Whether this is related to the fact that younger adolescents may have paid more attention or were more interested in the topic is not clear.

Perhaps the most reassuring finding is that students had good baseline knowledge about the danger of substance abuse during pregnancy, namely alcohol, cigarette and shisha smoking, and illicit drugs. This contrasts with other findings in Lebanon, where the prevalence of smoking in women during pregnancy is 25 to 27% [29] and the reported smoking rate in Lebanese adolescents is 60% [30]. However, recent aggressive campaigns against smoking [31] have led to the signature of a law on tobacco control (law 174), banning smoking in public places [32, 33]. Whether adolescents’ smoking behavior will change after the institution of this law remains to be explored, since knowledge does not necessarily translate into change in behavior.

This study highlights the need for increasing health literacy among adolescents early on during their school years and for targeting not only female but also male adolescents who are potential future partners in any pregnancy. The study also demonstrates that adolescents of all socio-demographic backgrounds should be targeted.

Although this intervention resulted in improved knowledge, several limitations are to be acknowledged. Time constraints and limited resources prevented the participation of 33.8% of chosen public schools; misconceptions and reservations regarding sensitive issues related to sexuality among adolescents were the reasons for refusal of participation by almost half of the private schools contacted. Furthermore, the intervention was relatively lengthy and was conducted in large classrooms, which limited the discussion time. The anonymous nature of the questionnaire also precluded pairing the pre- and post-test results for individual comparisons.

Finally, since the ministry of education recommended initiating this intervention in “girls only” public schools, there were only 18.9% male participants; this affects the generalizability of the findings in terms of gender differences.

Despite these limitations, this school-based intervention proved to be successful in improving preconception health knowledge. Yet, reaching large populations of students in classroom settings is time consuming and can hinder more detailed discussions. Reaching large numbers of adolescents in more efficient and appealing ways should be explored. Use of current technologies (e.g., mobile phones) to disseminate preconception health information to adolescents may be may be more appealing to them than getting information from parents, relatives or teachers.

## Conclusions

In summary, increasing knowledge and influencing behavioral change of young men and women is expected to improve the health of women before pregnancy and reduce non-life- threatening adverse outcomes (birth defects, low birthweight, prematurity). To this effect, using new approaches to include topics related to non-communicable diseases and reproductive health might prove to be influential; however, the feasibility, cultural acceptance and potential impact on knowledge and behaviors of this target population needs to be evaluated.

## Electronic supplementary material

Additional file 1: **Attached represents the pre and post knowledge assessment questionnaire used in this intervention.** (PDF 238 KB)

## Abbreviations

BT3: Technical Baccalaureate 3
CI: Confidence Interval
NCPNN: National Collaborative Perinatal Neonatal Network
NTD: Neural Tube Defects
RMNCH: Reproductive Maternal, Newborn and Child Health
SPSS: Statistical Program for Social Science.
