# Supplementary material for: Improving awareness of preconception health among adolescents: experience of a school-based intervention in Lebanon
Source: BMC Public Health. 2014 Jul 31;14:774. doi: 10.1186/1471-2458-14-774 (PMC4246486; doi:10.1186/1471-2458-14-774)
Supplement: Supplementary file 1 — Additional file 1: Attached represents the pre and post knowledge assessment questionnaire used in this intervention. (PDF 238 KB) [file 12889_2014_7278_MOESM1_ESM.pdf]

Appendix1:

Preconception health awareness intervention among adolescents in schools

National Collaborative Perinatal Neonatal Network (NCPNN)

This questionnaire is to evaluate your knowledge. Please respond to the questions by choosing one of the suggested answers. Do not choose the answers randomly; answer "don't know" if you are unaware of the correct answer.

DEMOGRAPHIC INFORMATION

1. School serial number: ________

2. Class: ☐ Grade 11 ☐ Grade 12

3. Age: _____ years

4. Gender: ☐ Male ☐ Female

5. Do you or any parent living in your house have any chronic condition (epilepsy, diabetes, heart disease, cancer etc)?
☐ Yes ☐ No ☐ Don't know

6. Father profession: _____________

7. Level of education of the father:
☐ Primary or less
☐ Complementary
☐ Secondary
☐ University

8. Do you have any married siblings (sisters or brothers)?
☐ Yes ☐ No

9. Are you engaged or planning to get engaged any time soon?
☐ Yes ☐ No

10. Mother profession: ___________

11. Level of education of the mother:
☐ Primary or less
☐ Complementary
☐ Secondary
☐ University

KNOWLEDGE ASSESSMENT

One answer per question

1. A women and her husband have decided to start trying to get pregnant. When should she schedule her first visit to the obstetrician?
☐ Right now, before she becomes pregnant
☐ As soon as she discovers she is pregnant
☐ After she's been pregnant for at least 3 months
☐ Don't know

2. At what age there is an increased risk of having a baby with trisomy 21 or Down syndrome:
☐ Less than 16
☐ Higher than 35
☐ Less than 16 and higher than 35
☐ Don't know

3. Which of the following might be effective in reducing the risk of birth defects?
☐ Iron
☐ Folic acid (vitamin B9)
☐ Calcium
☐ Don't know

4. If a diabetic woman (high blood sugar) becomes pregnant without proper planning or sugar control, she is at an increased risk to have a:
☐ Baby born with birth defect
☐ Baby born with ear infection
☐ Don't know

5. Before getting pregnant, a women should have her vaccination updated:
☐ True ☐ False ☐ Don't know

6. Which animal can cause abortion or serious infection if the pregnant woman handles its litter (wastes)?
☐ Dog ☐ Cat ☐ Don't know

7. Which of the following affect(s) the baby if it is taken by the mother before pregnancy:
☐ Alcohol
☐ Cigarettes / Arguileh
☐ Illicit drugs (cocaine, Marijuana)
☐ All the above
☐ None of the above
☐ Don't know

8. Obese pregnant women are at increased risk for which of the following:
☐ Diabetes of pregnancy
☐ Miscarriage
☐ Having a baby with birth defect
☐ All the above
☐ None of the above
☐ Don't know

9. Some acne medications might harm the fetus during pregnancy.
☐ True ☐ False ☐ Don't know

10. Women with epilepsy can get pregnant:
☐ True ☐ False ☐ Don't know
